# Impact of Positive Interferon-Gamma Release Assay on IVF-ET Pregnancy Outcomes in Infertile Patients With Untreated Prior Tuberculosis: A Prospective Cohort Study

**DOI:** 10.3389/fmed.2021.749410

**Published:** 2021-11-16

**Authors:** Xiaoyan Gai, Hongbin Chi, Lin Zeng, Wenli Cao, Lixue Chen, Chen Zhang, Rong Li, Yongchang Sun, Jie Qiao

**Affiliations:** ^1^Department of Respiratory and Critical Care Medicine, Peking University Third Hospital, Beijing, China; ^2^Center of Reproductive Medicine, Peking University Third Hospital, Beijing, China; ^3^Clinical Epidemiology Research Center, Peking University Third Hospital, Beijing, China; ^4^Tuberculosis Department, Beijing Geriatric Hospital, Beijing, China; ^5^Information Center, Peking University Third Hospital, Beijing, China

**Keywords:** interferon-gamma release assay, tuberculosis, IVF-ET, pregnancy outcome, infertility

## Abstract

**Introduction:** Tuberculosis (TB) is a major infectious disease that seriously endangers human health and female reproduction. In our previous study, 10.4% of infertile patients preparing for *In vitro* fertilization and embryo transfer (IVF-ET) had prior pulmonary TB (PTB) as detected on chest X-ray (CXR) screening. Among them, 81.8% did not receive anti-TB treatment. It remains unclear whether infertile women with untreated prior PTB have latent TB infection (LTBI) and whether LTBI affects IVF-ET outcomes. In this study, we aim to analyze the relationship between LTBI and pregnancy outcomes following IVF-ET in patients with untreated prior PTB.

**Methods and Analysis:** We designed a prospective cohort study of 1,200 infertile women with CXR findings suggestive of old-healed untreated TB, who are preparing for IVF-ET. Patients with a history of active TB and anti-TB treatment will be excluded. Interferon-gamma release assay (IGRA) will be used in patients with CXR findings suggestive of old-healed untreated TB to construct a cohort of IGRA-positive and IGRA-negative patients. Participants will undergo IVF-ET. General information, including age, body mass index, infertility causes, and controlled ovarian hyperstimulation protocol, will be recorded. Participants will be followed up during pregnancy. The primary outcome is live birth. Secondary outcomes include the numbers of retrieved oocytes, high-quality embryo rate, clinical pregnancy, number of active TB cases during pregnancy, and miscarriage.

**Ethics and Dissemination:** The study was approved by the Ethics Committee of Peking University Third Hospital [approval number (2020)218-01; approval date: June 19, 2020]. The research results will be disseminated through scientific/medical conferences and published in academic journals.

**Trial Registration:** ClinicalTrials.gov; identifier: NCT04443283.

## Introduction

Tuberculosis (TB), an infectious disease caused by *Mycobacterium tuberculosis*, seriously affects human health (1, 2). According to a report by the World Health Organization (WHO) in 2020, the global TB incidence rate is ~130 per 100,000 (1). TB may affect the reproductive ability of women and is a common cause of infertility due to blockage of the fallopian tubes or through the involvement of endometrium or ovarian functions (3, 4). Early identification of active TB cases and active anti-TB treatment are essential. However, the onset of TB is insidious, even without symptoms, and some patients may present with “infertility.” *In vitro* fertilization and embryo transfer (IVF-ET) remains the most effective method of treating associated infertility (5).

Although IVF-ET is an effective method, it is necessary to be aware of the harmful effects of TB during pregnancy. Chest X-ray (CXR) is an essential examination before IVF-ET, and active TB should be identified and treated accordingly. Seven cases of miliary TB during pregnancy after IVF-ET have been reported in our previous article (6). Active TB during pregnancy can lead to abortion and increase maternal and neonatal mortality (7–10). Therefore, patients with active TB are not allowed to undergo IVF-ET before receiving a full course of anti-TB treatment. However, during the routine screening before IVF-ET, CXR often reveals signs of prior pulmonary TB (PTB) (6), including fibrous scars, induration, and calcification, due to host interaction during *M. tuberculosis* infection (11, 12). Moreover, this is particularly common in developing countries. Whether anti-TB treatment is needed for so-called “prior pulmonary TB” becomes a clinical dilemma. Moreover, the possible side effects of anti-TB therapy, as well as the delay in pregnancy planning, are also concerns faced by doctors. Recently, we retrospectively analyzed 14,254 infertile patients who had undergone IVF at Peking University Third Hospital in 2017, and 1,487 (10.4%) patients were revealed to have PTB on CXR; among them, 1,239 (81.8%) did not receive anti-TB treatment and were followed to determine pregnancy outcomes (13). The results showed that the untreated prior PTB group had significantly lower clinical pregnancy and live birth rates than the non-PTB group. Moreover, during the follow-up, one patient with CXR findings suggestive of old-healed untreated TB developed miliary TB during pregnancy, which eventually led to abortion (13). We speculate that anti-TB therapy for high-risk patients may contribute to improving pregnancy outcomes. However, further prospective studies are needed to explore the risk factors that may affect pregnancy outcomes in this specific patient population.

Latent TB infection (LTBI) is defined by a positive test for infection and no active TB (14, 15). For high-risk TB patients, timely recognition of LTBI and preventive anti-TB treatment are also important measures for TB prevention and control. The tuberculin skin test (TST) or interferon-gamma release assay (IGRA) is a valuable tool for diagnosing LTBI and identifying people most likely to benefit from treatment (i.e., screening before the start of anti-tumor necrosis factor-α treatment, pre-transplant, and so on). TST has traditionally been used to detect LTBI, but it is known to have limitations. Most notably, its specificity is affected by the Bacillus Calmette–Guérin (BCG) vaccine or non-tuberculous Mycobacterium infection. IGRA (the QuantiFERON-TB [QFT] Gold In-Tube assay and the T-SPOT.TB assay), which measures cellular immune responses to the TB-specific proteins early secreted antigenic target of 6 and 10-kDa culture filtrate protein, has been demonstrated to be as sensitive as or better than TST for diagnosing LTBI, with much higher specificity, and no cross-reaction with the BCG vaccine (16).

Current guidelines recommend screening for LTBI with a TST, IGRA, or both; many also recommend chest radiography. Uzorka et al. reviewed 25 different studies on LTBI and CXR and reported that ~15% of individuals with LTBI had specific lesions on CXR that possibly reflect prior PTB (17). In contrast, ~77.7% of patients with prior PTB based on CXR findings were LTBI-positive. Persons with inactive TB lesions on chest radiographs have a risk of developing progression to active TB, with an incidence of 2.0–13.6 per 1,000 person-years (18). Our previous study showed that the IGRA positivity rate was 75.5% in infertile women with untreated PTB on CXR (unpublished data), which was significantly higher than the average rate of 13–20% previously reported by a study conducted in rural China (19). It remains unclear whether infertile women with untreated prior PTB have LTBI and whether there is a difference in the pregnancy outcomes between those with or without LTBI, and whether prophylactic anti-TB treatment is necessary for LTBI detected prior to IVF-ET in infertile patients.

Here, we hypothesized that adverse IVF-ET pregnancy outcomes in infertile patients might be related to untreated IGRA-positive prior PTB. If confirmed, further scientific evidence is essential to help understand the effect of LTBI on IVF-ET outcomes to optimize the management of LTBI in infertile patients with untreated PTB and update the guidelines.

We designed a prospective cohort study of infertile women who will undergo IVF-ET. IGRA will be performed in patients with CXR findings suggestive of old-healed untreated TB. A cohort of IGRA-positive and IGRA-negative patients will be constructed. Pregnancy outcomes will be followed prospectively, and the relationship between LTBI and pregnancy outcomes will be analyzed.

## Methods and Analyses

### Study Design

**Figure 1:**
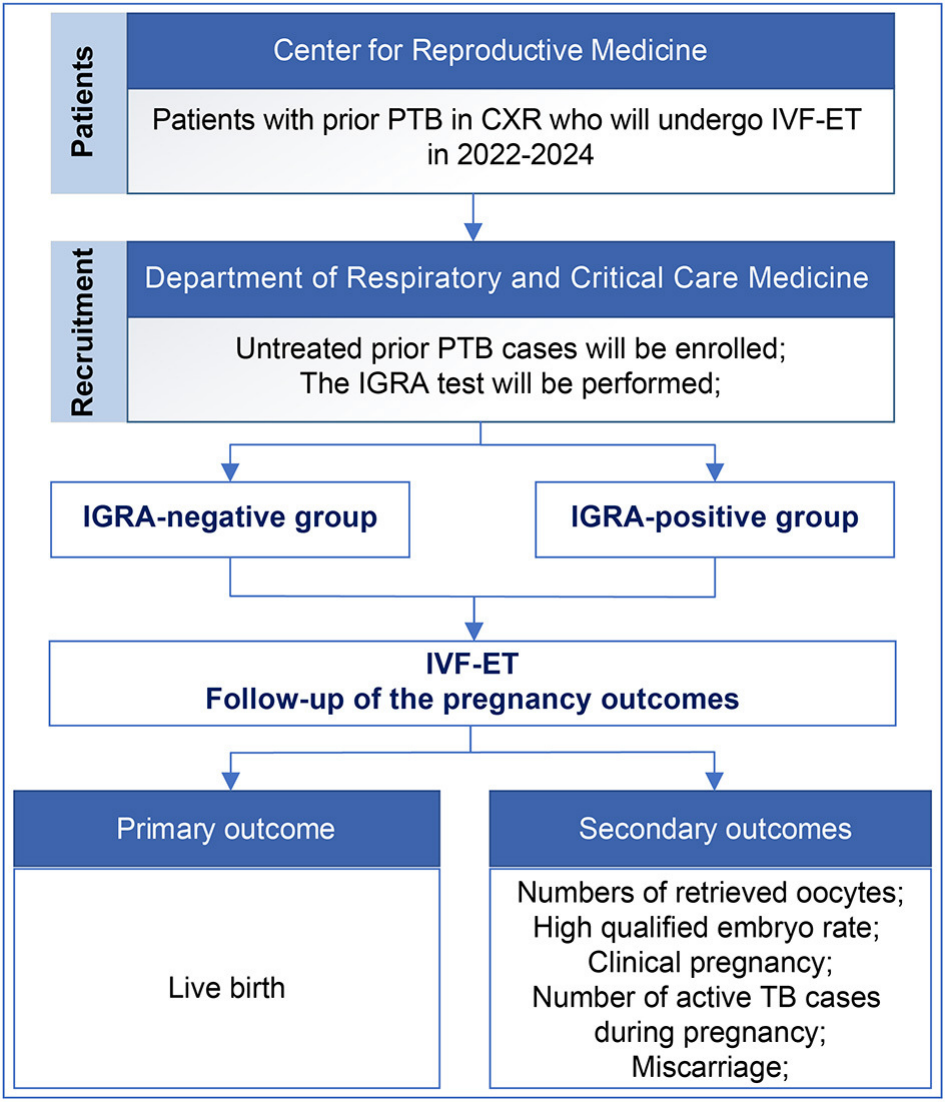
Flow chart of the study design. PTB, pulmonary tuberculosis; CXR, chest X-ray; IVF-ET, *In vitro* fertilization and embryo transfer; IGRA, interferon-gamma release assay.

This is a prospective cohort study of infertile patients who will undergo IVF-ET from January 1, 2022, to December 30, 2024, at Peking University Third Hospital. According to the sample size calculation, ~1,200 participants will be enrolled. The inclusion criterion is untreated PTB on CXR. The exclusion criteria are (1) active TB, (2) a previous diagnosis of TB, (3) TB other than PTB (e.g., reproductive system TB, renal TB, lymphatic TB), (4) the presence of central nervous system diseases, cancer, or AIDS, (5) long-term oral corticosteroids, immunosuppressants, and inhaled corticosteroid therapy, which may increase the risk of TB, and (6) patients who lack decisional capacity or who cannot guarantee their participation until the end of the study (Table 1). Participants will be divided into IGRA-positive and -negative groups, and the pregnancy outcomes will be followed during pregnancy. We will investigate whether LTBI (positive IGRA) in patients with untreated prior PTB affects the pregnancy outcomes (Figure 1).

**Table 1:** The inclusion and exclusion criteria for selecting the study participants.

**Primary inclusion criteria**	**Primary exclusion criteria**
• Age 18–45 years • Untreated prior PTB based on chest X-ray imaging findings	• Active TB•History of treated previous • TB other than PTB (e.g., reproductive system TB, renal TB, and lymphatic TB) • The presence of central nervous system diseases, cancer, or AIDS • Long-term oral corticosteroids, immunosuppressants, and inhaled corticosteroid therapy • Patients who lack decisional capacity or who cannot guarantee their participation until the end of the study

*AIDS, acquired immunodeficiency syndrome; PTB, pulmonary tuberculosis*.

### Procedures

Infertile patients who will undergo IVF-ET at the Center for Reproductive Medicine of Peking University Third Hospital will be screened. According to the Code of Practice for Assisted Reproductive Technology developed by the Ministry of Health of the People's Republic of China, all patients will undergo routine CXR examination before IVF-ET (20). Patients with abnormal chest images will be referred to the respiratory clinic for consultation, and those with chest imaging abnormalities consistent with prior PTB will be enrolled. Active TB cases will be excluded based on the clinical assessment (contact history, respiratory symptoms related to TB, physical examination), CXR, microbiological studies of sputum/respiratory samples, and endometrial biopsy findings. Moreover, among those with prior PTB based on CXR findings, patients will be excluded if they have a past history of treatment for active TB, and only those with untreated prior PTB will be enrolled. A total of 1,200 infertile patients with untreated prior PTB on CXR will be enrolled.

Participants' baseline characteristics (demographics, clinical characteristics, IVF-ET treatment) will be obtained from the case registration research database, including age, body mass index (BMI), infertility years, causes of infertility, and the controlled ovarian hyperstimulation (COH) protocol. Baseline levels of hormones will also be recorded, including follicle-stimulating hormone (FSH), luteinizing hormone (LH), estradiol (E2), progesterone (P), prolactin, testosterone (T), androstenedione (A), and anti-Müllerian hormone (AMH), and antral follicle count. Follicles after oocyte promotion, hormone levels (FSH, LH, E2, and P) on human chorionic gonadotropin (HCG) day, and B-ultrasound findings, such as a gestational sac and endometrial thickness, will also be recorded (Table 2).

**Table 2:** Baseline characteristics of the participants.

**General information**	**Age, race, body mass index, smoking, glucose**
Medical history	Primary infertility/secondary infertility; Infertility duration; Cause of infertility Male factors (oligospermia/asthenospermia/other male factors) Female factors (fallopian tube factor/polycystic ovary syndrome/endometriosis/other female factors) Unexplained infertility COH protocol Ultralong GnRH agonist; Short GnRH agonist; GnRH antagonist; Microsimulation cycle; Long GnRH agonist; Natural or other
Tuberculosis-related examination	Chest X-ray imaging Upper lung discrete linear or reticular fibrotic scars, sclerotic/calcified foci, and pleural thickening, with or without calcification of hilar/mediastinal lymph nodes. Erythrocyte sedimentation rate IGRA Salpingography Endometrial biopsy Laparoscopy, if available Hysteroscopy, if available
IGRA	IGRA positive or negative
Serum markers of ovarian function	Baseline hormone (Day 2–4 of menstruation) follicle-stimulating hormone (FSH), luteinizing hormone (LH), estradiol (E2), prolactin, testosterone (T), androstenedione (A), anti-Müllerian hormone (AMH), antral follicle count; Hormone levels (FSH, LH, E2, and P) on menstrual Day 2 of COH
Data of IVF-ET	No. of total sinus follicles No. of retrieved oocytes No. of good-quality embryos Endometrial thickness Endometrial pattern (A/B/C) No. of embryos transferred

*AMH, anti-Müllerian hormone; COH, controlled ovarian hyperstimulation; E2, estradiol; FSH, follicle-stimulating hormone; IGRA: interferon-gamma release assay; GnRH: gonadotropin-releasing hormone; LH, luteinizing hormone; No., number; P, progesterone; HCG, human chorionic gonadotropin*.

#### Follow-Up Plan

**Figure 2:**
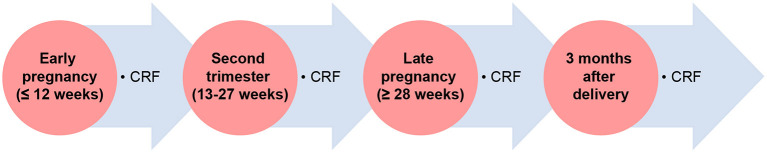
Follow-up of the pregnancy outcomes and time of assessment.

IVF-ET patients will be followed in early pregnancy ( ≤ 12 weeks), middle pregnancy (13–27 weeks), late pregnancy (≥28 weeks), and at 3 months after delivery. All pregnant women will be followed to fill out the case report form (CRF), and pregnancy outcomes will be assessed by telephone interviews (Figure 2).

##### CRF Information

Whether there are symptoms, such as fever, cough, night sweat, or blood in sputum during the pregnancy;

Whether blood routine test, chest X-ray examination, lung computed tomography examination, and sputum test have been performed and the results;

Whether the patient has been diagnosed with active TB;

Whether the TB has been diagnosed by clinical diagnosis, microbiology, or both;

Whether the pregnancy outcome is live birth, premature birth, abortion, or stillbirth; and

What are the birth weight and health statuses of the newborn?

#### LTBI Screening

LTBI testing will be conducted for the participants with untreated PTB using IGRA. The QFT assay (Qiagen, Hilden, Germany) will be performed according to the manufacturer's instructions. Individuals will be categorized into two groups based on the IGRA results: (1) Group 1: those with negative IGRA results and (2) Group 2: those with positive IGRA results. A special team will be assigned to perform follow-up on the pregnancy outcomes.

#### Interpretation of CXR

Prior PTB is defined as the presence of radiologic evidence suggestive of old/inactive PTB (11–13, 21, 22), including upper lung discrete linear or reticular fibrotic scars, sclerotic/calcified foci, and pleural thickening. This is an analysis of chest imaging findings, and a distinct history of TB is not necessary for the determination. Patients with other granulomatous diseases or tumors will be excluded. A chest radiologist and a respiratory clinician will confirm all CXR results.

#### IVF-ET Protocols

All the eligible participants will undergo a standardized COH protocol, oocyte retrieval, fertilization, and embryo transfer, as described previously (13, 23). COH will be followed by a planned transfer of one to two embryos (24). All embryos will be transferred on the third or fifth day after oocyte retrieval. The luteal-phase support with vaginal progesterone will be used from the day of ET until 10 weeks after conception. Clinical pregnancy is determined when an embryo sac is detected *via* ultrasonography at 30 days after ET.

### Primary Outcomes

The primary outcome is live birth (Table 3). Live birth is defined as the birth of at least one living fetus, and the live birth rate is calculated as live birth per embryo transfer.

**Table 3:** Overview of follow-up during pregnancy and time of assessment.

**Time**	**Case report form**
Early pregnancy ( ≤ 12 weeks)	1. Follow-up pregnancy outcome of the pregnant women live birth/premature birth/abortion/ stillbirth; 2. Whether there are symptoms of fever, cough, night sweat, or blood in sputum; If yes, a blood routine test, chest X-ray imaging, and lung CT examination will be performed. If pulmonary tuberculosis is diagnosed, it will be confirmed by clinical diagnosis/microbiology; 3. Follow-up of the newborn: birth weight and health status
Second trimester (13–27 weeks)	
Late pregnancy (≥28 weeks)	
Three months after delivery	

*CT, computed tomography*.

### Secondary Outcomes

The secondary outcomes include the numbers of retrieved oocytes, high-quality embryo rate, clinical pregnancy, number of active TB cases during pregnancy, and miscarriage (Table 3). Clinical pregnancy is determined upon observation of a gestational sac on ultrasonography. Miscarriage is defined as pregnancy loss before 28 weeks of gestation. The clinical pregnancy and miscarriage rates have been calculated as described earlier in this article (13, 23).

### Sample Size Calculations

We explored the effect of positive IGRA on pregnancy outcomes after adjustment for age, BMI, infertility causes, COH protocol, endometrial thickness, and number of good-quality embryos. There were 19 variables (including the dummy variables) in the logistic regression model. Considering the sample size requirement of multivariate logistic regression, 190 live birth events are required based on the 10 events per variable (including dummy variables) rule (25). Assuming that the live birth rate is 30% and the embryo transplantation rate is 53% (13, 23), a sample of 1,200 participants is required.

### Statistical Analysis

Statistical analysis will be performed using SPSS version 25 (IBM Corp., Armonk, NY). Continuous data will be expressed as means (± standard deviation), with independent sample *t*-tests or the Mann-Whitney U test for between-group differences. Categorical variables such as causes of infertility and COH protocol will be represented as frequency and percentage and analyzed using the Chi-square test.

The pregnancy outcomes of the IGRA-positive and IGRA-negative groups will be compared using the Chi-square test. We will use multivariate logistic regression to adjust for the effect of baseline characteristics. Adjusted odds ratios, 95% confidence intervals, and *p*-values will be reported. A two-tailed *p*-value < 0.05 will be considered statistically significant.

### Patient and Public Involvement Statement

This is an observational study. The patient will be informed of all the procedures and will be asked to sign the informed consent voluntarily.

## Discussion

To the best of our knowledge, this is the first prospective study on the impact of positive IGRA on IVF-ET pregnancy outcomes in infertile patients with untreated prior pulmonary TB. We will enroll 1,200 participants and analyze whether positive IGRA will affect the pregnancy outcome of IVF-ET. The primary outcome is live birth rate. Secondary outcomes include the numbers of retrieved oocytes, high-quality embryo rate, clinical pregnancy, number of active TB cases during pregnancy, and miscarriage.

Identifying and treating individuals with a high risk of progression from LTBI to active TB disease is critical. Studies have indicated that untreated participants with a positive IGRA result have a 10.8-fold higher rate of progression to active TB (26). Moreover, higher IGRA values are closely related to disease progression (27). Preventive anti-TB treatment is needed for LTBI in high-risk patients (15, 16); these patients include those with human immunodeficiency virus infection; those on immunosuppression therapy for tumors, necrosis factor α inhibitors, or glucocorticoids; or those receiving organ or hematologic transplantation (15, 16). However, there is no study on whether preventive anti-TB treatment is needed for infertile patients who will undergo IVF-ET. The so-called “prior” or “old” pulmonary TB lesion on CXR cannot be determined inactive or non-active lesion based on imaging alone. Our previous study showed that the clinical pregnancy and live birth rates of patients with untreated prior pulmonary TB, which was detected by CXR, after IVF-ET were lower than those without prior pulmonary TB on CXR, especially in those with unexplained infertility (13). In addition, reactivation of TB during pregnancy may cause abortion and great harm to pregnant women and fetuses. In our previous study, we included cases of miliary TB during pregnancy and found that most patients (6/7) had untreated prior pulmonary TB detected by CXR screening before IVF-ET (unpublished data). Identifying high-risk groups of TB and how preventive anti-TB intervenes before pregnancy are worthy of attention.

Our hypothesis is that LTBI will affect pregnancy outcomes in infertile women receiving IVF-ET. Knowledge of the relationship between LTBI and pregnancy outcomes could provide evidence for clinical decision-making regarding anti-LTBI treatment in this population. If this hypothesis is verified, further studies will be carried out to evaluate anti-TB therapy for patients with LTBI. The standardized protocol of isoniazid with rifampicin for 3 months may be administered for these infertile patients with LTBI, and improvements in pregnancy outcomes will be explored in the future.

### Strengths and Limitations

The current study has several key strengths. First, our prospective study design, in which LTBI and pregnancy outcomes are reliably followed, will allow us to analyze the relationship between LTBI and pregnancy outcomes and provide stronger evidence for causality. The results of our study can offer insights into LTBI and IVF-ET outcomes within a country with an intermediate TB burden, such as China. The outcomes of our study will serve as strong evidence for preventive anti-TB treatment of LTBI in infertile women within a country with an intermediate TB burden and provide data for updating guidelines. Second, this is a prospective study designed by a multidisciplinary team of respiratory physicians, obstetricians, obstetrician-gynecologists, epidemiologists, statisticians, and other related experts, a collaborative effort to ensure the quality of the study.

The limitation of the study is that this is a single-center study in China, without multi-center participation. However, as one of the largest and most authoritative reproductive centers in China, we perform nearly 20,000 IVF-ET cycles annually. Moreover, the population in this study is from all over the country and is representative of the Chinese population. In addition, accepted standards are available for IGRA quality control and the IVF-ET process in the same single center.

## Ethics Statement

The studies involving human participants were reviewed and approved by Ethics Committee of Peking University Third Hospital. The patients/participants provided their written informed consent to participate in this study.

## Author Contributions

RL and YS jointly conceived and directed this work, and RL is the leading corresponding author. XG and HC were responsible for protocol editing and recruitment of participants. LZ was responsible for sample size estimation and statistical analysis. YS and RL were involved in manuscript editing. All authors participated in the project and the acquisition, analysis, or interpretation of the data, and the final version has been reviewed and approved.

## Funding

This work was supported by the National Natural Science Foundation [No. 81400041 and 81871212], the National Natural Science Foundation of China Youth Fund Project [No. 81400038], and the Cohort study project of the Peking University Third Hospital (Y70545-04). The funding bodies had no role in the study design; in the collection, analysis, and interpretation of data; in the writing of the protocol; or in the decision to submit the article for publication.

## Conflict of Interest

The authors declare that the research was conducted in the absence of any commercial or financial relationships that could be construed as a potential conflict of interest.

## Publisher's Note

All claims expressed in this article are solely those of the authors and do not necessarily represent those of their affiliated organizations, or those of the publisher, the editors and the reviewers. Any product that may be evaluated in this article, or claim that may be made by its manufacturer, is not guaranteed or endorsed by the publisher.
